# Comparison between the effects of ultrasound guided intra-articular injections of platelet-rich plasma (PRP), high molecular weight hyaluronic acid, and their combination in hip osteoarthritis: a randomized clinical trial

**DOI:** 10.1186/s12891-022-05787-8

**Published:** 2022-09-12

**Authors:** Farshad Nouri, Marzieh Babaee, Parya Peydayesh, Hadi Esmaily, Seyed Ahmad Raeissadat

**Affiliations:** 1 Physical Medicine and Rehabilitation Research Center, School of Medicine, Shahid Beheshti University of Medical Sciences, Tehran, Iran; 2 Physical Medicine and Rehabilitation Research Center, Shahid Beheshti University of Medical Sciences, Tehran, Iran; 3 Physical Medicine and Rehabilitation Center and Department, School of Medicine, Shahid Beheshti University of Medical Sciences, Tehran, Iran; 4 Department of Clinical Pharmacy, School of Pharmacy, Shahid Beheshti University of Medical Sciences, Tehran, Iran; 5 Clinical Research Development Center, Shahid Modarres Hospital, Physical Medicine and Rehabilitation Research Center and Department, School of Medicine, Shahid Beheshti University of Medical Sciences, Saadat Abad St. Yadegare Imam Highway, Tehran, 1998734383 Iran

**Keywords:** Hip osteoarthritis, Intra-articular injections, Hyaluronic acid, Platelet-rich plasma, Ultrasound guided injection

## Abstract

**Background:**

Intra articular (IA) injection of platelet-rich plasma (PRP) and hyaluronic acid (HA) are of the new methods in the management of hip osteoarthritis (OA). The aim of this study was to compare the effectiveness of IA injections of PRP, HA and their combination in patients with hip OA. HA and PRP are two IA interventions that can be used in OA in the preoperative stages. Due to the different mechanisms of action, these two are proposed to have a synergistic effect by combining.

**Methods:**

This is a randomized clinical trial with three parallel groups. In this study, patients with grade 2 and 3 hip OA were included, and were randomly divided into three injection groups: PRP, HA and PRP + HA. In either group, two injections with 2 weeks’ interval were performed into the hip joint under ultrasound guidance. Patients were assessed before the intervention, 2 months and 6 months after the second injection, using the visual analog scale (VAS), Western Ontario and McMaster Universities Osteoarthritis Index (WOMAC), and Lequesne questionnaires.

**Results:**

One hundred five patients were enrolled randomly in HA, PRP and PRP + HA groups. All three groups showed significant improvement in WOMAC, VAS, and Lequesne at 2 months and 6 months compared with baseline. Comparison of the 3 groups demonstrated significant differences regarding WOMAC and Lequesne total scores and the activities of daily living (ADL) subscale of Lequesne (*P* = 0.041, 0.001 and 0.002, respectively), in which the observed improvement at 6th month was significantly higher in the PRP + HA and PRP groups compared to the HA group.

**Conclusion:**

Although all 3 interventions were associated with improvement of pain and function in patients with hip OA, the therapeutic effects of PRP and PRP + HA injections lasted longer (6 months), and the effects of these two interventions on patients’ performance, disability, and ADL were superior to HA in the long run. Moreover, the addition of HA to PRP was not associated with a significant increase in the therapeutic results.

**Trial registration:**

The study was registered at Iranian Registry of Clinical Trials (IRCT) website http://www.irct.ir/, a WHO Primary Register setup, with the registration number of IRCT20130523013442N30 on 29/11/2019.

## Introduction

The hip joint is the second large joint that most commonly affected by osteoarthritis (OA). The prevalence of hip OA increases with age and weight. It is also more common in those with lower physical activity [1]. This disease severely affects the quality of life of patients, while imposing a heavy financial burden on the society [2]. Since general life expectancy has been increased, and considering the high prevalence of this disease, more efforts has been made towards finding least invasive as well as inexpensive methods for the treatment of hip OA [3]. According to the 2013 guidelines of the American Academy of Orthopedic Surgeons (AAOS), the non-invasive treatments of OA include physical therapy, aerobic exercise, weight control, and neuromuscular education [4]. Moreover, paracetamol, non-steroidal anti-inflammatory drugs (NSAIDs) and intra-articular (IA) interventions such as corticosteroids (CS), hyaluronic acid (HA), and platelet-rich plasma (PRP) can be used as pharmacologic interventions [5].

IA injection of HA reduces the symptoms and pain of OA, while improving the function [6]. It does so by improving the lubrication of the joint, with minimal side effects [7, 8]. In addition to its viscoelastic properties, it stimulates the synthesis of endogenous HA and proteoglycans by chondrocytes, preventing the destruction of cartilage and lowering the level of inflammatory cytokines and matrix metalloproteinases in the synovial fluid. HA is one of the prior treatment methods, which has been approved by the food and drug administration for OA in 1997 and suggested by the American college of rheumatology (ACR) as a pain-reduction method in 2000 [9]. Yet, due to the studies regarding HA injections being biased towards the positive effects of this modality, the clinical outcomes have not shown these effects to be as good as expected, nor have the outcomes been similar in all studies. Because of this, since 2013, the AAOS has not recommend HA injections in OA patients who have symptoms [10].

On the other hand, these treatments are not very effective in preventing the destructive process nor in facilitating reconstruction. Therefore, there has been a tendency towards biological treatments, which have gained popularity in the recent years and have shown particularly good results in knee OA [11–15]. One of these treatments is PRP, which is prepared from centrifuging autologous blood to 2–5 times the normal platelet (PLT) concentration [16]. PLTs contain granules with abundant growth factors, which have roles in angiogenesis, tissue regeneration, chondrocyte proliferation, and cartilage matrix secretion [17], while also reducing the catabolic effect of interleukins which play a role in OA [18]. In various studies, PRP has been administered using different protocols in a variety of diseases [19, 20]. In a another study on the use of PLT rich fibrin, PRP, and plasma rich in growth factors (PRGF); the necessity of creating a standard protocol for the preparation of these products, explaining their exact PLT and growth factor content as well as long-term patient follow up has been pointed out [21].

Compared to knee OA, few studies have worked on the efficacy and comparison of PRP and HA injection in the hip joint; and in some of these studies, despite symptom improvement in both groups, no statistical significance has been observed between the two methods [22]. Some studies suggest that HA significantly reduces the pain and other symptoms in a long-term manner, with PRP having short-term effects [23]. In a study by Dallari et al., the results demonstrated further improvement in the PRP group and that the addition of HA did not alter the outcome and findings [24].

In view of the paucity of studies and the controversy that exists between the effectiveness and comparison of PRP and HA injections in hip OA, the current study was designed aiming to assess the effect of ultrasound (US) guided intra-articular PRP injection on pain reduction and the functional improvement of patients, using the visual analog scale (VAS), Western Ontario and McMaster Universities Osteoarthritis Index (WOMAC), and Lequesne questionnaires, and comparing its effects with HA as well as their combination in patients with hip OA.

## Patients & methods

### Design & setting

The study was registered at an online WHO primary register setup on November 29, 2019, with the registration code of IRCT20130523013442N30, a randomized clinical trial was conducted with in accordance with consolidated standards of reporting trials (CONSORT) guideline. The study was conducted in an outpatient clinic of physical medicine and rehabilitation at the Modaress Hospital; a teaching hospital affiliated with Shahid Beheshti University of Medical Sciences, Tehran, Iran. The hospital is a large referral center with a high patient turnover.

### Eligibility

We included men or women with grade two or three of hip OA, if they were 50–70 years, and with a duration of symptoms of more than 3 months in three parallel, equal-sized arms. The diagnosis of OA was based on x-ray imaging and ACR criteria and the grading was done based on Kellgren and Lawrence (KL) classification system. The exclusion criteria were; systemic diseases such as diabetes, immunodeficiency, collagen-vascular and autoimmune disorders, cardiovascular disease, active cancer or its history, an infection or wound in the hip region, severe deformity of the hip joint, PLT and bleeding disorders, use of NSAIDs from 1 week before injection, the patient being under treatment with antiplatelet or anticoagulant drugs during the past 10 days, having any intra-articular injection in the hip during the past 6 months or a systemic CS during the last 2 weeks, a hemoglobin level lower than 11 g/dL, PLTs less than 150 × 10 [3]/μL, history of recent severe trauma to the hip, hypersensitivity to HA, history of using blood thinning herbs, supplements or vitamins 2 weeks before injection, obese patients with body mass index of more than 30 kg/m^2^ and neurogenic claudication in favor of spinal stenosis.

### Recruitment

At first, patients with hip OA were invited to attend a screening visit. The study phases and rationale were explained to all potential participants during the interview in the first visit. If a patient declined to participate, another was selected and invited in the same way until the needed sample had been recruited. At the screening visit past medical history, physical examination, laboratory findings, including; C-reactive protein (CRP), erythrocyte sedimentation rate (ESR), complete blood count (CBC), and a standing A-P view pelvic x-ray obtained. If it is necessary, a lumbosacral magnetic resonance imaging and electrodiagnostic studies, were requested. The patients’ drug history and supplements use were asked and recorded in case report forms complies with good clinical practice principles. We reviewed documents and the patients were then presented to a consensus committee of the authors who confirmed their eligibility and invited them to participate in the study. Participants who gave written informed consent, were randomly allocated to one of the study groups.

### Interventions

At the beginning of the study, information regarding PRP and HA injection as well as their benefits and possible side effects were presented both orally and in written form by a physical medicine and rehabilitation specialist. In all groups, the intervention was performed with two injections 2 weeks apart.

In the first group, 5 ml of autologous PRP was injected, in the second group, 2.5 ml injection contained 50 mg linear fermentation source high molecular weight HA was injected (Viscor 50 mg/2.5 mL, molecular weight of 2500–3200 kDa, Nitka, Iran). In the third group, first, 5 ml of PRP and immediately afterwards, 2.5 ml of HA was injected.

### Preparation of PRP

First, 35 ml blood was taken from the antecubital vein using an 21G needle. Afterwards, 5 ml of acid citrate dextrose solution containing 2.20-g sodium citrate dehydrate and 0.73-g sodium citrate anhydrous plus 2.45-g dextrose monohydrate was added as an anticoagulant. A single milliliter of the blood was sent to the lab for CBC and leukocyte differentiation. The storage temperature of the bloods was normal room light and temperature. The PRP processing was done using a registered standard kit (Rooyagen kits, Arya Mabna Tashkhis corporation, registration number: 312569). Samples were put into four test tubes and centrifuged for 12 minutes at 1600 rpm (RPM) equal to 400 g based on relative centrifugal force in first stage as light spin, which resulted in three different layers. The lowest layer was RBC precipitate, the middle layer was white blood cell (WBC) s, and the upper layer was plasma. The plasma containing PLT, together with the buffy coat layer was slowly aspirated and transferred to two test tubes in order to be centrifuged in second stage at 3500 RPM (= 1900 g) for 7 minutes as heavy spin. In the final stage, after aspiration and disposal of PLT poor plasma, a sample of PRP sent for PLT and WBC counts, 5–6 ml of liquid PRP (around 3 ml in each tube) with at least 4 times of whole blood PLT count was approved for injection. The optimum processing time targeted to be 2 h after blood collection. Processed PRPs stored at room temperature/light and were injected into the hip joints after a shaking with standard tube shaker without any additive (activator or cytokine), stem cell and scaffold within maximum 4 hours from blood collection. For standard reporting, minimum information for studies evaluating biologics in orthopedics (MIBO) checklist for PRP and mesenchymal stem cells studies was followed.

### Intra-articular injection technique

**Fig. 1:**
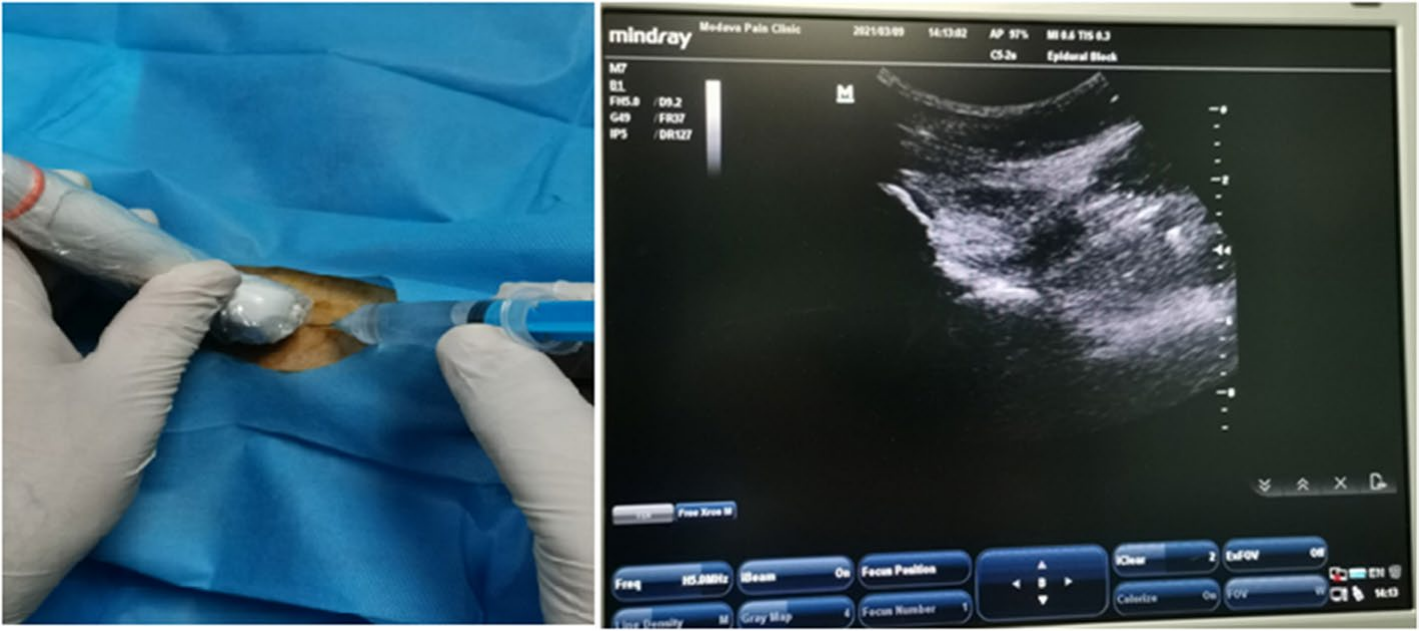
Injection practice under US guidance

The injection was performed in the hip joint under US guidance, using the classic approach. For this, in all three groups, the patients were put in the supine position and after preparation and draping of the injection site, in sterile conditions and under US guidance, the hip injection was performed. A 23G (blue) spinal needle was inserted into the anterior capsular recess, between the neck and head of the femur, in a caudocranial and lateromedial manner (Fig. 1). In all three groups, the patient was allowed to leave after 10–15 minutes of rest. The second injection was performed 2 weeks later under similar conditions.

In all three groups, the patients were sent home with written instructions. They were instructed not to take a bath or shower for 24 h, have relative rest for 24-48 h, with minimal weight-bearing and use aiding devices such as a cane or crutches. A cold compress three times a day for 10 minutes was recommended. Patients were allowed to use paracetamol 500 mg (without codeine) every 8 hours and increase the dose up to every 4 hours if the pain was not controlled. Paracetamol plus codeine was given if the patient needed further pain control.

The patients were not allowed to use any other pain relief medication such as NSAIDs, PLT-affecting medications, blood thinning herbs, supplements or vitamins for 4 days after injection as well as steroids for 1 week after injection. It was generally suggested that they continue low to medium physical activity and gradually increase it at their own pace.

Exercise therapy was recommended for every participant, the protocol of which consisted of isometric exercise of the muscles around the hip as well as mild stretching exercises 3 times a day, with 10 repetitions for each move for 10 seconds. After 1 month, closed chain-isotonic exercises were added to the training program.

### Outcome measures & follow-ups

The primary outcome of this study was WOMAC total score and the secondary outcomes was VAS, score of the Lequesne questionnaire and patient satisfaction as well as complications of injection at 6 months.

In this study, the participants were evaluated three times: before the intervention, 2 months, and 6 months after the second injection. The tools used were VAS, the WOMAC and the Lequesne questionnaire.

### Western Ontario and McMaster universities osteoarthritis index (WOMAC)

The Persian version of WOMAC was filled through an interview with a physical medicine and rehabilitation resident, and included 24 questions in three categories (pain, stiffness, and functional limitations). A lower sum of points meant less pain and better function [25].

### Visual analogue scale (VAS)

The VAS is for pain evaluation and ranges from 0 (no pain) to 10 (severe pain). The participants were asked to show the maximal pain they had experienced during the last 2 days on the VAS ruler.

### Lequesne questionnaire

The Lequesne questionnaire is an eleven-part questionnaire designed for obtaining subjective information regarding the hip joint. From these 11 parts, five are related to pain, and discomfort while staying in a certain position or situation, or performing a particular movement; two asks about the maximum walking distance and use of walking aids, while the remaining four concerned daily functional abilities. In this tool a higher score is indicative of higher disability [26].

### Patient satisfaction & complications of injection

All patients were assessed regarding complications such as stiffness, heaviness, pain and their treatment satisfaction based on a 5-point Likert scale consisting; 1) Very Dissatisfied, 2) Dissatisfied, 3) Neutral, 4) Satisfied and 5) Very Satisfied.

### Sample size

In, Dallari et al. study 111 participants were divided in three groups of 36 patients in HA, 44 in PRP and 31 one in the combination group, and measured some outcomes, including pain in VAS, WOMAC and Harris hip score at baseline, 2, 6 and 12 months post intervention [24]. Their results showed that at the baseline the two groups were similar in the WOMAC. However, at 6 months there was a significant difference between HA and PRP groups in the mean WOMAC index; 59 [95% CI, 54–65] versus, 72 [95% CI, 67–76] *P* = 0.009, respectively, and the difference was not significant between HA group and the combination group. SD, combined SD and effect size of 0.366 were calculated using validated formulas [27, 28]. Considering this effect size, for the ability to detect a significant discrepancy in WOMAC index between groups at 6 months, a power of 80%, and a two-tailed *P*-value (*P*) of 0.05 as statistically significant, the calculated total sample size was 78 participants. Due to the occurrence of coronavirus disease of 2019 (COVID-19) pandemic after the start of the recruitment, concerns about increasing the number of drop out patients of the study increased, therefore the researchers decided to allocate 24 more patients in the study to maintain 80% power in case of increased drop out to 30%, hence 105 patients were enrolled in the study.

### Randomization & blinding

For the random allocation of participants to three groups with the same size of 35 participants (105 patients in total), we used an online tool to create a blocked randomization list with 7 blocks of 15 samples with three treatment groups [29]. The random numbers were generated in an independent statistical room. The allocation sequence was hidden for all investigators and participants with sequentially numbered sealed envelopes which contained cards with the assignment type. Opening of the envelopes and preparation of the injection solutions, and the injection were done by an expert physiatrist, with more than 12 years’ experiences in IA injection to hip joint, who were neither involved in the allocation nor the assessments.

Due to the fact that more blood was drawn from participants in the PRP and PRP + HA groups, it was not possible to completely blind the patients. All research team decided to give the participants a same explanation for the amount and frequency of blood transfusions and the purpose of the blood draw, so that patients would not be informed by the care providers about which group they were in. However, all follow-up assessments were done by blinded investigators.

### Statistical analyses

The collected data was kept in each patient’s profile and was analyzed using statistical package for the social sciences (SPSS) version 24. For the comparison of normally distributed data, T-test, analysis of variance (ANOVA) and for non-normal distribution, the Wilcoxon and Kruskal–Wallis test were employed. Qualitative data were analyzed using Chi-square test. In order to assess the interaction effects of time and group on the outcome measures, repeated measures analysis of variance (ANOVA) was used as well as post hoc complementary tests for within/between subjects’ analysis and for within groups pairwise comparison of results, paired-samples T test were applied. The level of significance was determined as less than 0.05 in this study.

## Results

**Fig. 2:**
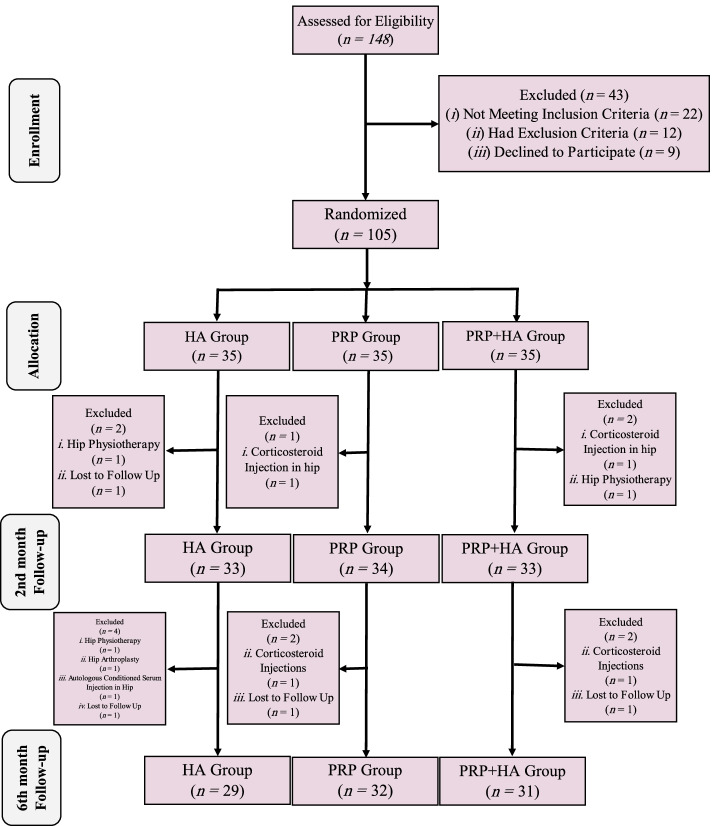
Clinical trials flow diagram

The study recruitment began on April 6, 2019, the date of entry of the last patient into the study was September 14, 2019, and the study data gathering was completed on March 16, 2020. A total of 105 patients with mild to moderate (grade 2 to 3) hip OA were entered into this study. The participants were randomly assigned to three groups of HA (35 patients), PRP (35 patients), and PRP + HA (35 patients). In the HA group 6 patients, in the PRP group 3 patients, and in the PRP + HA group, 4 patients left the study during follow ups, eventually, 92 patients were finalized the study per protocol, Fig. 2, shows the clinical trial flow diagram in more details.

Within the 92 patients, 25 were male (27.2%) and 67 were female (72.8%). There was no meaningful difference among the three groups in demographic variables as well as the WOMAC, VAS, and Lequesne results or their sub-categories before the study (*P* >  0.05) (Table 1).

**Table 1:** Participants’ demographics and baseline evaluations

Characteristic		HA	PRP	PRP + HA
Number		29	32	31
**Baseline Characteristics**				
Age (*y*), *Mean ± SD*		60.93 ± 4.54	58.22 ± 5.10	60.29 ± 4.83
Sex (male/female), *#*		7/22	10/22	8/23
Hip OA grade (2/3), *#*		16/13	16/16	17/14
Involved hip (right/left), *#*		12/17	20/12	17/14
Pain duration (*month*), *Mean ± SD*		3.43 ± 1.53	4.63 ± 2.50	4.26 ± 2.03
Height (*m*), *Mean ± SD*		1.65 ± 0.08	1.67 ± 0.10	1.65 ± 0.09
Weight (*kg*), *Mean ± SD*		74.93 ± 6.30	77.63 ± 10.05	76.03 ± 8.36
BMI (*kg/m*^*2*^), *Mean ± SD*		27.62 ± 2.25	27.72 ± 2.11	27.94 ± 2.80
CBC	Hb (*g/dL), Mean ± SD*	14.3 ± 1.52	13.59 ± 1.78	13.54 ± 1.70
	WBC, (*× 10*^*3*^*/*μL*), Mean ± SD*	6.34 ± 1.40	6.45 ± 1.12	6.23 ± 1.07
	PLT, *(× 10*^*3*^*/*μL*)*, *Mean ± SD*	269.51 ± 61.07	246.90 ± 9.25	235.83 ± 47.93
History of physiotherapy (> 1 month) (yes/no), *#*		13/16	23/9	18/3
History of previous injection (> 6 months) (No/PRP/HA/CS), *#*		21/2/5/1	16/7/6/3	18/6/5/2
**Outcome Measures,*Mean ± SD***				
WOMAC	Pain	9.28 ± 1.41	9.53 ± 1.72	9.68 ± 1.49
	Stiffness	2.38 ± 1.21	2.75 ± 1.83	2.71 ± 1.01
	Function	30.41 ± 8.72	29.09 ± 7.09	28.77 ± 6.84
	Total	41.41 ± 11.52	41.38 ± 9.36	41.16 ± 8.13
VAS		8.10 ± 1.18	7.63 ± 1.31	8.00 ± 1.18
Lequesne	Pain	5.45 ± 1.02	4.91 ± 1.25	5.16 ± 1.10
	MDW	1.79 ± 1.05	1.56 ± 0.67	1.55 ± 0.57
	ADL	5.59 ± 0.71	5.63 ± 0.76	5.74 ± 0.67
	Total	12.52 ± 2.34	12.20 ± 2.18	12.45 ± 1.66

The quality control of PRP characteristics showed, PRP samples had a 5.5 ± 1.09 times more PLT counts than the whole blood samples, with 70–90% PLT recovery rate and an average counts of 2.19 ± 0.37 × 10 [3]/μL WBCs. Table 2, shows the PRP characteristics in more details.

**Table 2:** PRP characteristics

PRP Characteristics, ***Mean ± SD***				
Group (#)		PRP (32)	PRP + HA (31)	*P*-value
PLT Count (×10^3^/μL)		1402.03 ± 387.58	1240.35 ± 294.23	.068
PRP/Blood PLT Ratio		5.71 ± 1.24	5.29 ± 0.87	.131
WBC Count (× 10^3^/μL)		2.21 ± 0.39	2.16 ± 0.37	.587
Leukocyte Differential Count	Lymphocyte (×10^3^/μL) (%)	1837.40 ± 333.32 82.81 ± 1.65	1778.74 ± 308.25 82.19 ± 1.72	.471
	Neutrophil (× 10^3^/μL) (%)	243.34 ± 44.69 11.00 ± 0.88	245.16 ± 46.29 11.32 ± 0.70	.875
	Monocyte (× 10^3^/μL) (%)	136.03 ± 46.04 6.18 ± 1.89	140.09 ± 43.45 6.48 ± 1.80	.720

*PLT* Platelet, *WBC* White blood cell, *PRP* Platelet-reach plasma

Among the post-injection complications, only pain after injection was significantly different among the three groups, with the HA group experiencing less pain after injection (1.68 ± 0.92) compared to the PRP (3.50 ± 2.22) and PRP + HA (3.22 ± 2.40) groups (*P* = 0.001). Regarding other complications, 17 patients from all three groups experienced complications such as warmness, stiffness, and heaviness. Between the three groups, no significant difference was observed (*P* = 0.873). Regarding the amount of patient satisfaction after injection, from the 92 participants, 54 of them were either satisfied or very satisfied with the procedure. The highest amount of dissatisfaction was among the HA group, and the lowest was seen in the PRP + HA group. Overall, there was not any meaningful difference regarding patient satisfaction between the groups (*P* = 0.838).

In Table 3, the outcome measurement results of WOMAC, VAS and Lequesne as well as their domains are shown at baseline, 2 months, and 6 months after the intervention. All outcomes show meaningful improvement in all three groups compared to baseline at 2 months and 6 months after.

**Table 3:** The changes of outcome measures

	Outcome Mean ± SD	Before intervention	After 2 months		After 6 months		Group and Time Interaction
			Values	*P*-value	Values	*P*-value	
**WOMAC Pain**	PRP + HA	9.68 ± 1.49	4.55 ± 1.59	< 0.001	4.52 ± 1.84	< 0.001	0.058
	PRP	9.53 ± 1.72	4.63 ± 1.86	< 0.001	4.59 ± 1.83	< 0.001	
	HA	9.28 ± 1.41	4.79 ± 1.50	< 0.001	5.45 ± 1.66	< 0.001	
**WOMAC Stiffness**	PRP + HA	2.71 ± 1.01	1.03 ± 0.95	< 0.001	0.97 ± 0.91	< 0.001	0.676
	PRP	2.75 ± 1.83	1.28 ± 1.22	< 0.001	1.03 ± 1.26	< 0.001	
	HA	2.38 ± 1.21	1.00 ± 1.00	< 0.001	1.00 ± 0.96	< 0.001	
**WOMAC Function**	PRP + HA	28.77 ± 6.84	17.19 ± 6.10	< 0.001	15.68 ± 6.16	< 0.001	0.299
	PRP	29.09 ± 7.09	17.66 ± 6.17	< 0.001	15.91 ± 7.96	< 0.001	
	HA	30.41 ± 8.71	19.38 ± 6.89	< 0.001	19.93 ± 6.90	< 0.001	
**WOMAC Total**	PRP + HA	41.16 ± 8.13	22.78 ± 7.44	< 0.001	21.16 ± 8.00	< 0.001	0.041
	PRP	41.38 ± 9.36	23.56 ± 8.18	< 0.001	21.53 ± 10.40	< 0.001	
	HA	41.41 ± 11.52	25.38 ± 8.61	< 0.001	27.21 ± 9.25	< 0.001	
**VAS**	PRP + HA	8.00 ± 1.18	2.48 ± 1.03	< 0.001	3.13 ± 1.18	< 0.001	0.359
	PRP	7.63 ± 1.31	2.38 ± 1.07	< 0.001	3.13 ± 1.29	< 0.001	
	HA	8.10 ± 1.18	2.69 ± 1.11	< 0.001	3.90 ± 1.40	< 0.001	
**Lequesne Pain**	PRP + HA	5.16 ± 1.10	3.58 ± 1.36	< 0.001	3.06 ± 1.31	< 0.001	0.160
	PRP	4.91 ± 1.25	3.53 ± 1.32	< 0.001	3.13 ± 1.54	< 0.001	
	HA	5.45 ± 1.02	3.66 ± 1.20	< 0.001	3.83 ± 1.31	< 0.001	
**Lequesne MDW**	PRP + HA	1.55 ± 0.57	1.06 ± 0.44	< 0.001	1.23 ± 0.67	0.041	0.546
	PRP	1.56 ± 0.67	1.13 ± 0.22	0.003	1.28 ± 0.73	0.010	
	HA	1.79 ± 1.05	1.21 ± 0.67	< 0.001	1.31 ± 0.71	0.001	
**Lequesne ADL**	PRP + HA	5.74 ± 0.67	4.24 ± 1.18	< 0.001	3.79 ± 1.37	< 0.001	0.001
	PRP	5.63 ± 0.76	4.38 ± 1.26	< 0.001	4.09 ± 1.16	< 0.001	
	HA	5.59 ± 0.71	4.25 ± 0.81	< 0.001	4.78 ± 0.87	0.002	
**Lequesne Total**	PRP + HA	12.45 ± 1.66	8.89 ± 2.50	< 0.001	8.08 ± 2.55	< 0.001	0.002
	PRP	12.20 ± 2.18	9.09 ± 2.73	< 0.001	8.59 ± 2.99	< 0.001	
	HA	12.52 ± 2.34	9.34 ± 2.04	< 0.001	10.29 ± 2.82	0.002	

When comparing the outcome measures and the changes in their domains among the three groups at the time between the 2nd and 6th months post-intervention, statistically significant difference was observed only in the total WOMAC score, activities of daily living (ADL) from the Lequesne questionnaire, and the total Lequesne score. This means that the changes in these given times were different between the three groups. Table 4 shows pairwise comparisons of outcomes in details.

**Table 4:** The comparison of outcome measures in different time periods

	Outcome	Baseline compared to 2nd month		Baseline compared to 6th month		2nd month compared to 6th month	
		MD (95% CI)	Multiple Comparison	MD (95% CI)	Multiple Comparison	MD (95% CI)	Multiple Comparison
**WOMAC Total**	PRP + HA	18.39 (14.89–21.88)	PRP + HA vs. PRP: NS	20.00 (15.96–24.05)	PRP + HA vs. PRP: NS	1.61 (−0.92–4.15)	PRP + HA vs. PRP: NS
	PRP	17.81 (14.37–21.25)	PRP + HA vs. HA: NS	19.84 (15.86–23.83)	PRP + HA vs. HA: 0.007	2.03 (− 0.46–4.52)	PRP + HA vs. HA: 0.020
	HA	16.03 (12.42–19.65)	PRP vs. HA: NS	14.21 (10.03–18.39)	PRP vs. HA: 0.022	−1.83 (−4.45–0.79)	PRP vs. HA: 0.021
**Lequesne ADL**	PRP + HA	1.50 (1.03–1.97)	PRP + HA vs. PRP: NS	1.95 (1.42–2.49)	PRP + HA vs. PRP: NS	0.45 (0.07–0.83)	PRP + HA vs. PRP: NS
	PRP	1.25 (0.79–1.71)	PRP + HA vs. HA: NS	1.53 (1.00–2.06)	PRP + HA vs. HA: < 0.001	0.28 (− 0.09–0.65)	PRP + HA vs. HA: 0.012
	HA	1.33 (0.85–1.81)	PRP vs. HA: NS	0.81 (0.26–1.37)	PRP vs. HA: < 0.001	− 0.52 (− 0.91 – − 0.13)	PRP vs. HA: 0.001
**Lequesne Total**	PRP + HA	3.57 (2.61–4.52)	PRP + HA vs. PRP: NS	4.37 (3.36–5.38)	PRP + HA vs. PRP: NS	0.81 (0.10–1.52)	PRP + HA vs. PRP: NS
	PRP	3.05 (2.10–3.99)	PRP + HA vs. HA: NS	3.55 (2.55–4.54)	PRP + HA vs. HA: < 0.001	0.50 (− 0.20–1.20)	PRP + HA vs. HA: < 0.001
	HA	3.17 (2.18–4.17)	PRP vs. HA: NS	2.22 (1.18–3.27)	PRP vs. HA: 0.027	−0.95 (− 1.68 – − 0.21)	PRP vs. HA: 0.012

*MD* Mean Difference, *95% CI* 95% Confidence Interval, *PRP* Platelet-Rich Plasma, *HA* Hyaluronic acid, *NS* Non-significant, *WOMAC* Western Ontario and McMaster Universities Osteoarthritis Index, *ADL* Activity of Daily Living

**Fig. 3:**
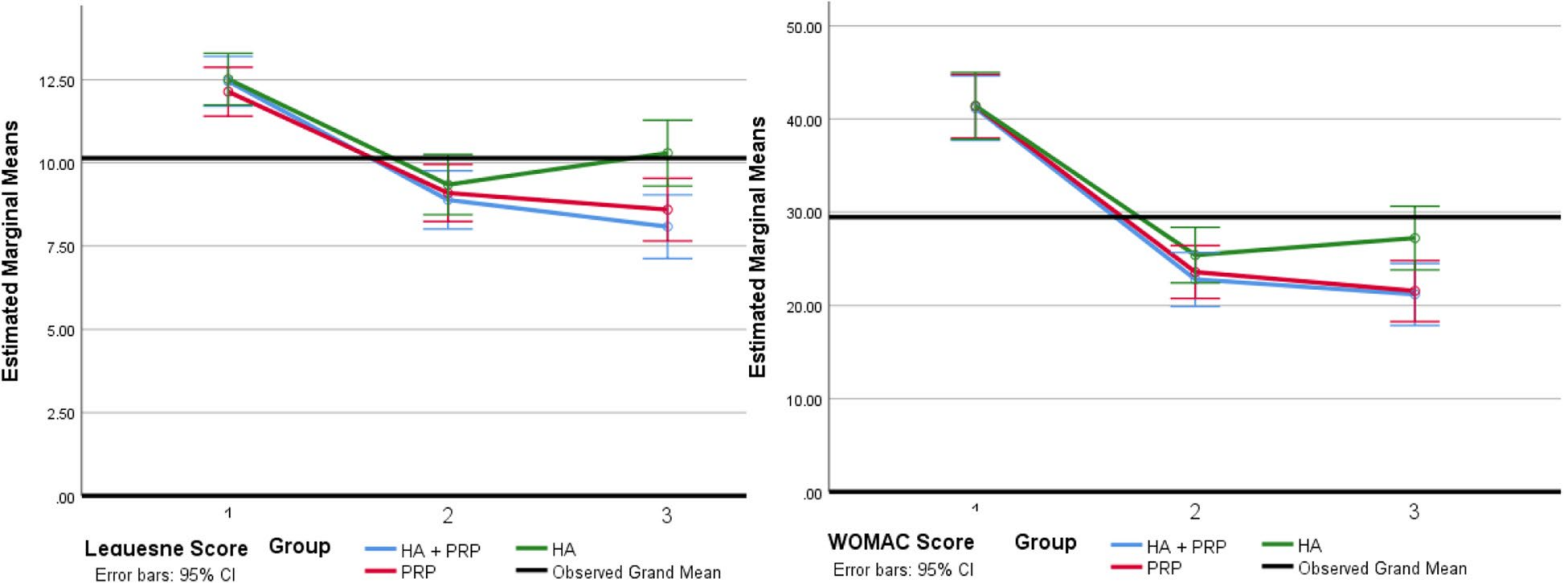
Changes of overall Lequesne and WOMAC score

Considering the mean difference of variables and comparing all three groups with each other in these given times, it was observed that the average changes among both the PRP and PRP + HA group were better than the HA group (Table 5, Fig. 3).

**Table 5:** Participants with 30% or more than 30% recovery in VAS, WOMAC, and Lequesne, 2 and 6 months follow up

	Outcome	PRP + HA Number (%)	PRP Number (%)	HA Number (%)	*P*- value
2 months follow up	WOMAC Total	26 (83.9)	26 (81.3)	24 (82.8)	0.963
	VAS	30 (96.8)	30 (93.8)	29 (100)	0.390
	Lequesne Total	11(35.5)	10 (31.3)	13 (44.8)	0.563
6 months follow up	WOMAC Total	23 (74.2)	23 (71.9)	19 (65.5)	0.749
	VAS	29 (93.5)	28 (87.5)	25 (86.2)	0.616
	Lequesne Total	19 (61.3)	13 (40.6)	6 (20.7)	0.006
VAS [P-value]		WOMAC [P-value]	Lequesne Total [P-value]		
PRP + HA vs. PRP [0.672]		PRP + HA vs. PRP [> 0.999]	PRP + HA vs. PRP [0.133]		
PRP + HA vs. HA [0.417]		PRP + HA vs. HA [0.576]	PRP + HA vs. HA [0.002]		
PRP vs. HA [> 0.999]					

*PRP* Platelet-Rich Plasma, *HA* Hyaluronic acid, *VAS* Visual Analogue Scale, *WOMAC* Western Ontario and McMaster Universities Osteoarthritis Index

According to Tables 5, the percentage of participants whose improvement in total WOMAC, VAS and total Lequesne scores was 30% or more (success rate) only showed a significant difference among the three groups in total Lequesne score after 6 months. This means that compared to the HA group, more people from the PRP + HA group had 30% or higher improvement after 6 months and this difference was meaningful. There was not any similarly significant difference found between the PRP and PRP + HA groups.

## Discussion

According to the findings of the current study, all three interventions have led to improvement in pain and function of patients with hip OA and all three groups have shown significant differences in WOMAC, VAS and Lequesne at 2 and 6 months after injections compared to the beginning of intervention. While comparing the process of change between the three groups (between 2 and 6 months), only the total WOMAC score, ADL from the Lequesne questionnaire, and the total Lequesne score showed a significant difference. This difference is indicative of the long-term superiority of the effect of PRP and combined injections over HA on function, disability, and ADL. On the other hand, the addition of HA to PRP added no meaningful benefit to the intervention.

Compared to knee OA, a small number of studies have compared the effect of HA and PRP for symptom management in hip OA. Zhao et al. [30] in their meta-analysis in 2020, compared the effect of various intra-articular injections (Cs, PRP, HA, PRP + HA) on hip OA from 11 studies. In this study, intra-articular injection of CS was the best short term (1 month) treatment for pain relief and improvement in function. At 3 months, according to WOMAC, PRP + HA showed the best results. In the long term (6 months), PRP demonstrated the best effect on pain reduction. In our study results, similar to the Zhao et al. study, no significant difference was found between the groups at 2 months, while at the 6-month assessment, the PRP group showed better pain relief. Our study at this time (6 months) though, showed better total WOMAC and Lequesne in both PRP and PRP + HA groups compared to the HA group.

In a meta-analysis by Garcia et al. in 2020, 7 studies regarding the role of PRP on hip disorders (femoroacetabular impingement syndrome (FAIS), labral pathology, and OA) were assessed [31]. Among the four studies evaluating PRP in HIP OA, the most important finding was not seeing any statistically significant difference between HA, PRP, and PRP + HA in hip OA treatment after 1 year. Although pain reduction and outcome improvement of PRP injection in hip OA does continue 12 months after injection, these effects are more evident in the first 4–6 months; and it seems, its therapeutic effects begin to diminish after that. Nevertheless, this meta-analysis does not show a meaningful difference between the pain reduction of PRP and HA (based on VAS) 1 year after injection. These findings are almost similar to ours, showing no significant long-term differences in pain reduction between groups.

Comparing these two systematic review and meta-analyses with each other and also the current study, while considering of the similarities and differences in the findings, it seems that the number of studies entered into the meta-analysis, the presence or lack of a control group, and the employment of various outcome measures has led to heterogeneity in the analyses. Therefore, the final results are not similar in some aspects, requiring further studies in this field.

The theory of combining PRP and HA in OA in humans was pioneered by Andia and Abate. Based on the opinions of the writers and the studies performed in the laboratory on animal models, which show the synergistic effect of HA and PRP, it was suggested that combined therapy may be more effective. According to these studies, HA and PRP can affect the joints’ cells through independent mechanisms and facilitate cellular signals such as inflammatory molecules, catabolic enzymes, cytokines, and growth factors. This can aid in repairing degenerated cartilage and delaying the process of OA, and play a positive role in the treatment of knee OA. This synergistic effect often changes the role of inflammatory cytokines in the destruction of chondrocytes through specific mediators (CD44، TGF-βRII), leading to cartilage regeneration as well as the inhibition of the inflammatory response [32]. According to the meta-analysis and review by Kumar et al. [33] and Gilat et al. [34], PRP and HA may have synergy and despite limited data, a combination of PRP + HA may clinically improve pain and function of the patients with knee OA.

Using a combination of PRP + HA in the management of hip OA has been studied in the current study and the study of Dallari et al. [24]. Based on the findings of these two studies, combined use of HA and PRP does not lead to a meaningful improvement in symptoms compared to PRP alone. Considering the low number of studies in this regard, more randomized controlled trial (RCT) s on the combination of these substances in hip OA are necessary.

Compared to other RCTs which were mostly used in meta-analyses [22–24, 35, 36], the results suggesting pain reduction and outcome improvement mostly concern the earlier months post-injection, gradually after which the PRP and PRP + HA groups maintain their effects as time goes on. In studies where PRP showed no superiority of over HA, the main reason seems to have been the higher age of the participants and entering patients with higher grades of OA into the study. Therefore, the important point in the choice of the time of injection, is the choice of the patient, in the sense that patients of a lower age group with lower degrees of OA benefit more from PRP injections. Studies in recent years have reported that clinical improvements in PRP injections are time-related and on average are sustained for about 9 months, while having better and longer lasting results with lower amounts of articular degeneration [37]. Studies have shown that the effect of HA diminishes with time, particularly in older patients [38, 39]. HA mainly nourishes, lubricates, and protects the joints; and has a lower effect on the joint repair and regeneration processes. Due to its high content of growth factors, PRP can reinforce chondrocyte production and cartilage matrix synthesis [23], which can lead to longer lasting effects [32]. The findings show the lower effect of PRP on older patients and those with higher stages of joint degeneration. In more severely degenerated joints, a lower percentage of viable cells exist, reducing the response to growth factors. All these ideas are based on guesses and theories, which need to be proven through studies designed to understand the relationship between age and cartilage degeneration in response to PRP injection [23].

In addition to age and higher OA stages affecting study results, the difference in the PRP preparation protocol and its contents, and the number of PRP injections have all shown to play a role in the results and findings, which has also been one of the challenges in meta-analyses [30, 31].

Even though systematic reviews and meta-analyses of knee OA have suggested pain reduction and functional improvement via PRP injections, due to the differences in cartilage structure and biomechanics, it is not correct to generalize their results to the hip joint. Despite this, PRP appears to be better than HA at controlling the symptoms of patients with hip OA, and this is more evident in studies with longer follow up periods (6 months to 1 year).

This study is one of the few which assess HA, PRP, and PRP + HA together, while evaluating three separate outcome measures. The biggest drawback of this study is the inability of proper blinding of the patients and doctors due to the nature of the substances used, which can lead to some bias. Furthermore, the volume of injection in each group was not equal, which could also act as a confounding factor. The other limitation is the lack of a negative control (sham) group such as a saline or lidocaine injection. In addition, in the current study, patients were only followed for 6 months. In view of the effects of the injections being time-dependent, longer follow up could have led to a better understanding of the role of PRP and its sustained effects. Also it is suggested to measure objective outcomes beside subjective variables in the future studies.

In this study we used a linear fermentation based high molecular weight HA (~ 3000 kDa), considering varieties in IA injections of HA, differing in concentration, linear or cross-linked and source of the HA, it needs other studies to investigate the efficacy and safety of other products.

With the findings of the current study in mind, although all three interventions showed pain reduction and functional improvement, the therapeutic effects of PRP as well as combined treatment lasted longer (6 months), and the effects of improving function, disability, as well as ADL are superior to HA in the long run. Furthermore, adding HA to PRP is not resultant in any meaningfully better therapeutic effects.

## Data Availability

The datasets generated and/or analyzed during the current study are available in the https://drive.google.com/drive/folders/1KML5P4GUJ2xLw9B5vP1O7k6EqGD20l1U, also the data will be sent if a request is sent to the corresponding author.

## Abbreviations

AAOS: American Academy of Orthopedic Surgeons
ACR: American college of rheumatology
ADL: Activities of daily living
ANOVA: Analysis of variance
CBC: Complete blood count
CONSORT: Consolidated standards of reporting trials
COVID-19: Coronavirus disease of 2019
CRP: C-reactive protein
ESR: Erythrocyte sedimentation rate
FAIS: Femoroacetabular impingement syndrome
HA: Hyaluronic acid
Hb: Hemoglobin
IA: Intra articular
KL: Kellgren and Lawrence
MIBO: Minimum information for studies evaluating biologics in orthopedics
NSAIDs: Non-steroidal anti-inflammatory drugs
OA: Osteoarthritis
PLT: Platelet
PRGF: Plasma rich in growth factors
PRP: Platelet-rich plasma
RPM: Revolutions per minute
SPSS: Statistical package for the social sciences
US: Ultrasound
WBC: White blood cell
WOMAC: Western Ontario and McMaster Universities osteoarthritis index
